# Is a downwards head tilt a cross-cultural signal of dominance? Evidence for a universal visual illusion

**DOI:** 10.1038/s41598-021-04370-w

**Published:** 2022-01-10

**Authors:** Zachary Witkower, Alexander K. Hill, Jeremy Koster, Jessica L. Tracy

**Affiliations:** 1 Department of Psychology, University of Toronto, 100 St. George Street, Toronto, ON M5S 3G3 Canada; 2 University of Washington, Seattle, USA; 3 University of Cincinnati, Cincinnati, USA; 4 University of British Columbia, Vancouver, Canada

**Keywords:** Psychology, Human behaviour

## Abstract

The present pre-registered research provides the first evidence that a downwards head tilt is sufficient to communicate dominance from a neutral facial expression among the Mayangna, members of an unindustrialized, small-scale traditional society in Nicaragua who have had minimal exposure to North American culture. Consistent with the Action Unit imposter effect observed in North American populations (Witkower and Tracy in Psychol Sci 30:893–906, 2019), changes to the appearance of the upper face caused by a downwards head tilt were sufficient to elicit perceptions of dominance among this population. Given that the Mayangna are unlikely to associate a downwards head tilt or related apparent facial changes with dominance as a result of cross-cultural learning, the present results suggest that perceptions of dominance formed from a downwards head tilt, and the visual illusion shaping these perceptions, are a widely generalizable, and possibly universal, feature of human psychology.

## Introduction

Over 50 years ago, researchers demonstrated that facial expressions associated with a small set of emotions are reliably recognized across a range of populations, including traditional small-scale societies that had little to no contact with the Western world^1,2^. Given that the preliterate, culturally isolated participants in some of these studies could not have learned about Western emotion expressions through cross-cultural transmission, these findings are widely considered to be one of strongest pieces of evidence supporting the case for universal emotions, and, more broadly, an evolved human nature (^3–8^, but see^9^).

Building on this seminal work, subsequent cross-cultural research on nonverbal communication has focused largely on facial expressions of emotion, yet nonverbal behaviors beyond the face are also regularly employed in human social interactions, and are used to communicate a variety of emotional and non-emotional messages. For example, studies have identified a distinct, cross-culturally recognized nonverbal display that includes the body as well as the face, and that functions to communicate *dominance* – a form of high social rank associated with the use of aggression and intimidation to elicit fear and forced deference (Witkower et al., under review;^10^. This display features bodily expansion, a neutral facial expression, and a downwards head tilt^10^. In North American samples, this display elicits perceptions of dominance even when observers view only the downwards head tilt and neutral face presented in isolation, devoid of any visible facial or bodily movement^11^.

To account for the strong signal of dominance sent by a downward-head tilt alone, Witkower and Tracy^11^ proposed the *action unit imposter* account, wherein tilting one’s head downward causes the eyebrows to take on an apparent V shape and seem to become lowered—the same appearance cues associated with Action Unit (AU) 4, the “eyebrow lowerer” (i.e., corrugator muscle activity), in Ekman and Friesen’s^11^ Facial Action Coding System (FACS). Numerous studies have found that AU4 is associated with facial expressions of dominance, threat, and anger across cultures^1,13–18^, and Witkower and Tracy^11^ found that these same social messages were communicated by a downward-head tilt and neutral face, even though no AU4 activity was present. In other words, by mimicking the appearance cues associated with AU4, tilting one’s head downward creates the illusory appearance of this action unit, and thereby conveys a similar social message. In this way, a downward-head tilt serves as an “imposter” of AU4.

Given that lowering one’s brow, or activating AU4, is a cross-cultural signal of threat and dominance, and a downwards head tilt causes the same appearance changes as this facial movement, a downwards head tilt might also be a cross-cultural signal of dominance. Prior studies have found that downwards head tilt conveys dominance among North American^19–21^ and Portuguese^22^ populations, but in all of these studies participants were Westernized, Educated, Industrialized, Rich, and Democratic (WEIRD,^5^). No prior research has tested whether a downwards head tilt communicates dominance across populations that are maximally divergent from WEIRD samples, and, if so, whether that is because it functions as an AU imposter across human societies—that is, whether this visual illusion is a widely generalizable and potentially universal feature of human psychology. Although we expected this effect to generalize across cultures, there is also reason to suspect it might not,other widespread visual illusions, such as the Muller-Lyer effect, which were long assumed to be universal features of human perception, are now known to be a consequence of unique features of WEIRD cultural learning (i.e., in the case of Muller-Lyer, “carpentered corners”; Segall et al. 1966;^5^). It therefore remains an open question whether the AU imposter effect is likely to be a human universal.

In fact, if a downward-head tilt is found to increase dominance perceptions across cultures, there are alternative explanations for this effect, beyond the AU imposter account. Previous scholars have suggested that a downwards head tilt might communicate dominance (or related constructs such as threat and intimidation) by changing the apparent dimensions of the head (i.e., facial width-to-height ratio;^19^, changing the apparent size of the chin^23^, making the head and neck appear closed and contracted relative to the body^24^, changing the appearance of the mouth^25^, Kappas et al. 1994), or defensively covering the neck with the chin^19^. Critically, all of these other visual mechanisms rely on parts of the head and face *besides* the eyebrows, whereas the AU imposter account is based on changes to the appearance of the eyebrows alone. Prior research on the AU imposter mechanism in North America systematically ruled out each of these alternatives^11^, but it remains unknown whether these mechanisms might account for any impact of downward-head tilt on dominance perceptions in other cultural contexts.

## The current study

In the present pre-registered research (see https://osf.io/sev3x/?view_only=9e3fdc9764774c08b7b9e2a3c27f8c11), we tested whether a neutral face with a downward-tilted head is perceived as dominant by the Mayangna – members of an unindustrialized, small-scale traditional society in Nicaragua. The Mayangna exist in a cultural context that is highly divergent from that examined in previous work on the AU imposter effect, and they have limited exposure to North American culture. As a result, if the Mayangna perceive a downwards head tilt as dominant, it would be highly unlikely that these perceptions are a result of acculturation. Findings along these lines would provide strong evidence to suggest that this head movement functions to evoke a signal of dominance that generalizes broadly across human populations, and might therefore be universal.

We also tested whether, if this effect emerged in this sample, it is likely to be due to the AU imposter illusion. To do so, we showed participants stimuli featuring the upper face only, with the rest of the face and head visually occluded. If a downwards head tilt increases perceptions of dominance by changing the appearance of the eyebrows (i.e., by serving as an imposter of AU4), participants should perceive a neutral face with a downwards tilted head as dominant even when all other facial and head features besides eyes and eyebrows are hidden—that is, when even the head tilt itself is not visible. In contrast, if a downwards head tilt increases perceptions of dominance by changing the appearance of the jaw, mouth, or facial height, or by contracting the head to cover the neck or bring it closer to the body, this behavior would *not* increase perceptions of dominance compared to a neutral head angle when participants view only the eyes and eyebrows in isolation. Therefore, by restricting visible stimuli to the narrow band of the face where the eyes and eyebrows are located, we can test whether the AU imposter mechanism operates cross-culturally, while pitting it against previously proposed visual mechanisms.

We preregistered two hypotheses: (1) among the Mayangna, a neutral face with downwards head tilt will increase perceptions of dominance compared to the same facial expression with a neutral head angle, and (2) this effect will emerge in this population even when the eyes and eyebrows are shown in isolation (https://osf.io/sev3x/?view_only=9e3fdc9764774c08b7b9e2a3c27f8c11).

## Method

### Participants

The Nicaraguan community sampled for this research was comprised of indigenous Mayangna horticulturalists living primarily in the forested region of the Bosawas Biosphere Reserve^26–29^. We aimed to recruit as many members of this community as possible,ultimately the sample included more than approximately 90% of the adult members of the community: 119 individuals (65 female) who ranged from age 18 to 75 (*M* age = 34.23; *SD* age = 14.62).

Several factors make it likely that these individuals have little familiarity with western global culture. First, only 15% indicated that they could read and write fluently in Spanish—the national language of Nicaragua. Overall, participants had little formal education (*M* = 6.0 years, *SD* = 4.12 years) and minimal direct exposure to western media (71% had never seen a US movie, 84% had never seen a US television program, and 94% had never used the internet). Nearly all participants had never left Central America (97%) or Nicaragua (95%). Roughly half (47%) reported leaving their village once per year or less, and the other half (49%) reported leaving the village roughly once per month. That said, some of these individuals might have experienced some minimal exposure to western culture as a result of meeting members of the current research team (or previous research teams), as well as sporadic visits from non-indigenous health officials, conservationists, or representatives. Older members of the community might have encountered American military personal during the Contra war in the 1980s.

All participants completed the present study after first participating in two separate studies, one examining recognition of dominance and prestige displays from full-body images (Witkower et al., under review), and the other examining emotion recognition (i.e., anger, fear, sadness) from body-only (i.e., face occluded) images^30^. These prior studies found that: (a) Mayangna participants reliably recognized expressions of dominance from full-body images featuring targets with an expansive bodily posture with their hands on the hips, their heads tilted downward, and neutral facial expressions^31^ and (b) Mayangna participants reliably recognized body-only expressions of anger, fear, and sadness (see^30^).

As a result of their involvement in these prior studies, these individuals had, prior to their participation in the current study, viewed other stimuli featuring a downwards head tilt as part of the dominance display. In both prior studies, however, displays were posed by human targets instead of a computer-generated avatar, so participants never saw the images presented here outside of this study. Although it is nonetheless possible that carry-over effects could influence results in the present study (e.g., participants might recall seeing full-body dominance displays and use the judgment they made in viewing them to infer dominance from the head-only images shown here), we see this possibility as unlikely, given how different the stimuli in both other studies were from those used here (i.e., in addition to being actual human posers, targets used in prior studies varied in ethnicity and gender; and in all prior study images, full bodies were shown rather than head or face only). Furthermore, across all three studies participants completed a total of 78 trials, comprised of comparisons among 28 different images, thus reducing the likelihood that they would remember their response to any particular image seen previously.

Given our goal of recruiting individuals who are unlikely to possess considerable knowledge about Western or global popular culture, we first assessed participants’ familiarity with global popular culture by asking them to identify images of 13 celebrities: Donald Trump, Barack Obama, Hillary Clinton, Oprah Winfrey, Will Smith, Brad Pitt, Taylor Swift, Lebron James, Lionel Messi, Cristiano Ronaldo, Michael Jordan, Elvis Presley, and Abraham Lincoln. To assess participants’ exposure to the culture of industrialized Nicaragua, we also showed them an image of Daniel Ortega, the current President of Nicaragua who served as head of state in non-concurrent terms for 22 of the 40 years preceding data collection. For each image, participants were asked “Who is this?” and responded aloud in an open-ended fashion. On average, participants correctly identified fewer than one of the 13 popular cultural icons (*M* = 0.54 images, *SD* = 0.86, Mode = 0; Range = 0 to 3), and 67% correctly identified Ortega. In comparison, among a sample of 273 American MTurk workers, participants correctly identified an average of 82% of the 13 popular culture icons (*M* = 10.60, *SD* = 1.83, Mode = 11, Range = [0,13]), and only 2% recognized Ortega. These results suggest that Mayangna participants had minimal knowledge of western or broader global popular culture. It is noteworthy, however, that they had modest familiarity with the culture of more industrialized parts of Nicaragua. Therefore, as a more stringent test of our hypothesis, we planned to conduct additional exploratory analyses on the subset of participants who failed to recognize any of the 13 global icons and also failed to recognize the current President of Nicaragua.

### Materials and procedure

**Figure 1:**
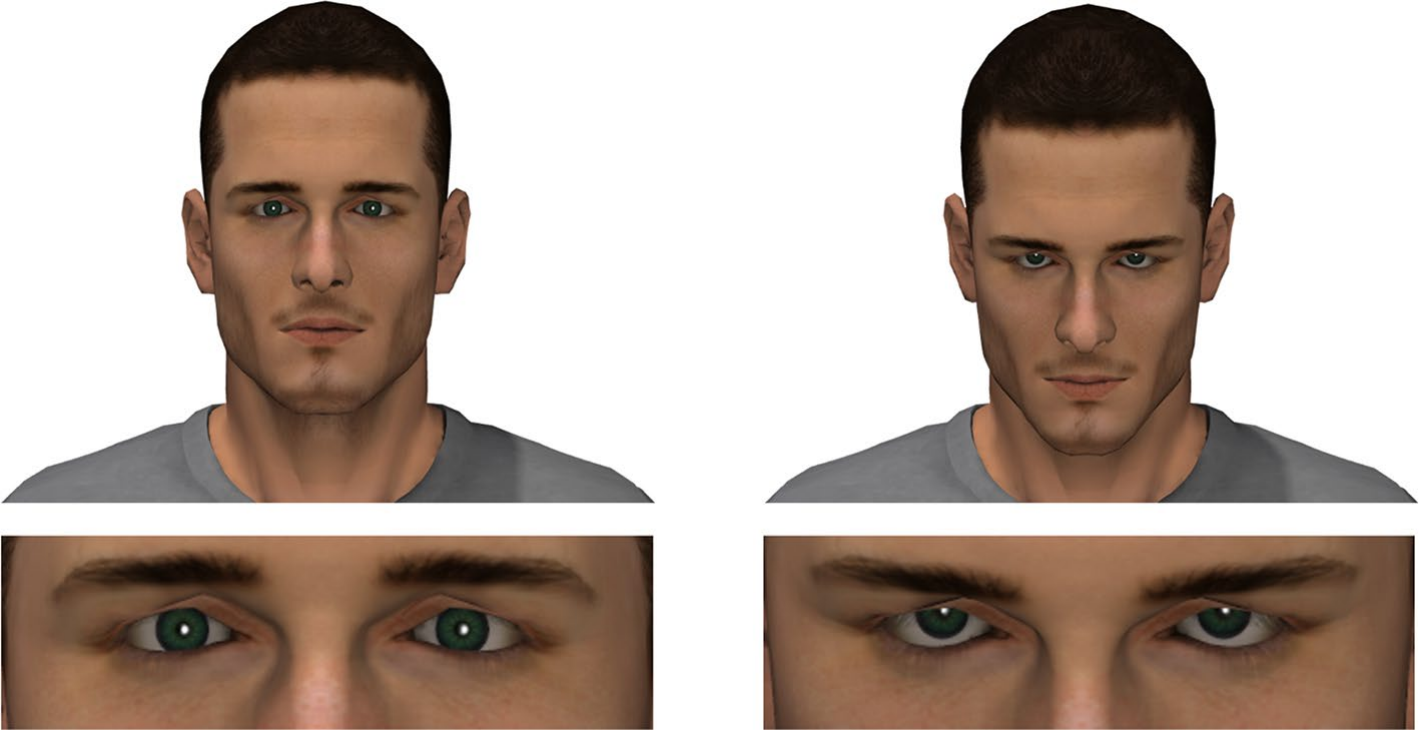
Neutral head angle (left) and downward-head tilt (right) stimuli, with the whole head visible (top), and the upper face visible with the rest of the face and head occluded (bottom). Stimuli were generated using Smith Micro^32^ Poser Pro computer software.

A computer-generated male avatar created in past research^11^ was used in the current study (see Fig. 1). Stimuli were generated using Smith Micro^32^ Poser Pro computer software. Using an avatar allowed us to precisely manipulate the target’s head angle while preventing any incidental facial movements. The avatar was portrayed with eye gaze directed towards participants and his head at either a neutral angle (0°) or tilted down (10°; see Fig. 1). Although a 10° tilt is subtle, this small behavioral change has been found to effectively promote perceptions of dominance in prior work^11^, and is consistent with the kinds of behaviors that tend to occur during real-life social interactions.

In each of two trials, participants were shown two images side-by-side, contrasting a neutral head angle with a downwards head tilt (with eye gaze directed towards the observer in both cases). In the first trial, participants viewed the stimuli featuring the upper face only (i.e., the narrow band from the cheekbones to the brow ridge, excluding the forehead and mouth; see Fig. 1), to test whether the AU imposter mechanism is responsible for any effect of downward-head tilt on perceived dominance. In the second trial participants viewed the whole head, to test whether a downward-head tilt is sufficient to communicate dominance with no additional bodily information available. By ordering the trials in this manner, we ensured that the ‘whole head’ stimuli did not influence perceptions formed from the upper face alone.

For each trial, participants were asked: “Please select the image in which the person is likely to be a leader because he is willing to use aggression and intimidation to get his way”. An English version of this prompt was previously validated to reliably assess dominance in U.S. samples (see Witkower et al., under review). All materials were translated from English to Spanish (and back-translated from Spanish to English) prior to the study, and then translated from Spanish to the Mayangna and Miskito languages on-site by co-author JK and two research assistants fluent in Spanish, Mayangna, and Miskito (for original materials in English, Spanish translations of those materials, and Spanish-to-English back-translations, see https://osf.io/ad6n2).

The study was performed according to ethical standards as laid down in the 1964 Declaration of Helsinki and 1979 Belmont Report, and was approved and reviewed by the University of Cincinnati Institutional Review Board. All individuals who participated in the current study also provided informed consent and permission to use their data after being formally briefed about the study.

## Results

**Figure 2:**
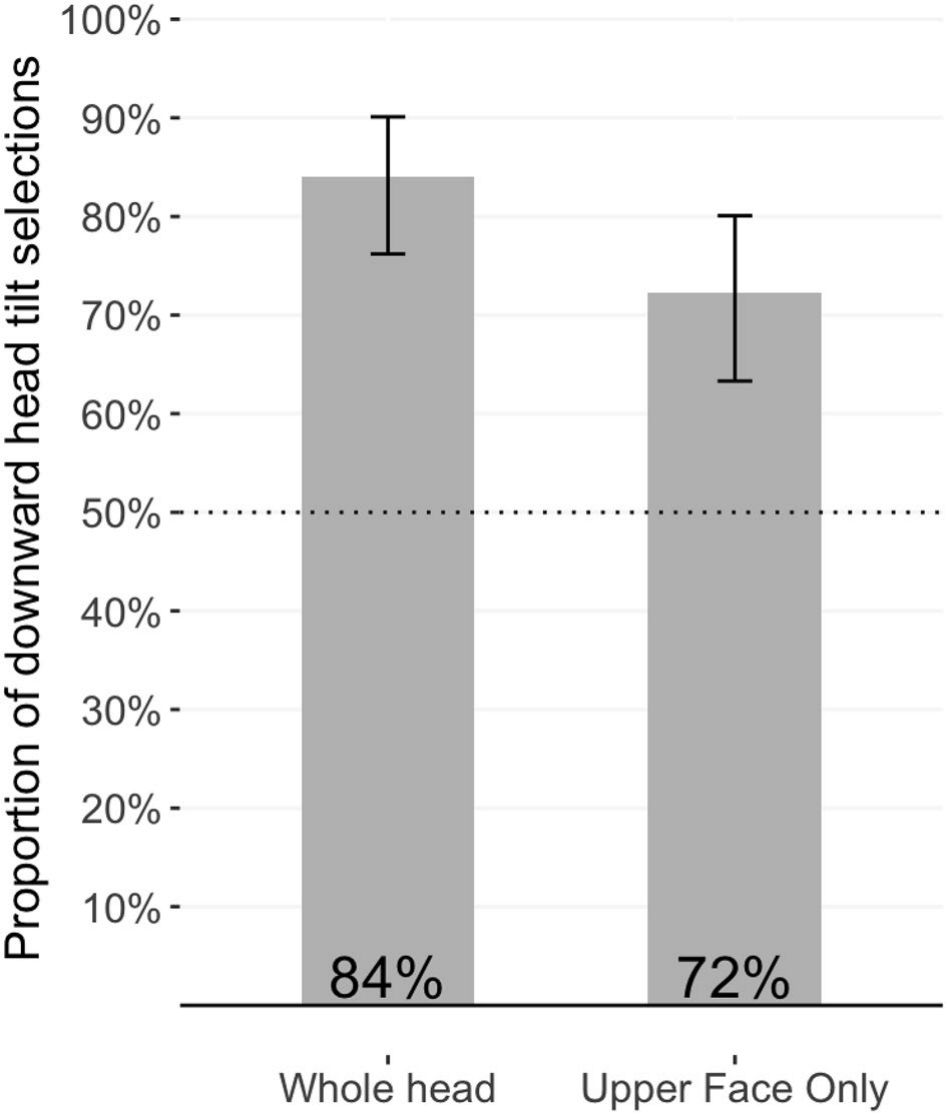
Proportion of downward-head tilt selections in response to the dominance prompt, when participants were shown the whole head (left), and the upper face in isolation (right).

In line with our pre-registered analysis plan, two binomial tests were conducted to test whether participants selected the downward-head tilted version of each expression as the more dominant image at rates significantly greater than chance (i.e., 50%, given that participants selected between two images). Consistent with our hypotheses, in both trials participants identified the image featuring a downwards head tilt as significantly more dominant than the image with a neutral angle head; for full-head images, 84%, *p* < 0.001, 95% CI [0.76 to 0.90]; for upper-face only images, 72%, *p* < 0.001, 95% CI [0.63 to 0.80] (see Fig. 2). These two rates differed significantly, *X*^2^(1) = 4.16, *p* = 0.04, suggesting that the information provided by the presentation of the full head contributed to dominance perceptions beyond what was gleaned from the upper face only. However, the small improvement in rates could also be due to order effects; participants might have become more confident in their perceptions of dominance upon seeing the critical image for a second time (immediately after the first), given that the full-head image contained all of the visual information present in the upper-face only image.

### Exploratory analyses

As a more stringent test of our hypothesis, we next analyzed data only for those participants who failed to recognize *any* of the 13 global icons, and also failed to recognize the current President of Nicaragua, who served as head of state in non-concurrent terms for 22 of the 40 years preceding data collection. This subsample, which we refer to as the *highly isolated* subsample, consisted of 36 individuals (25 female, *M* age = 37.86 years; *SD* = 16.89 years). Thirty-four of these 36 participants (63%) reported leaving their village once a year or less, and seven had never left their village.

Consistent with findings from our prior pre-registered analyses, participants in the highly isolated subsample identified images featuring a downwards head tilt as more dominant than images featuring the neutral head angle, when viewing the full head, 78% *p* < 0.001, 95% CI [0.61 to 0.90], and when viewing the upper face only, 69%, *p* < 0.001, 95% CI [0.52 to 0.84]. These two rates did not differ significantly, *X*^2^(1) = 0.29, *p* = 0.59.

## Discussion

The current study demonstrates that a downwards head is sufficient to communicate dominance among the Mayangna, and that changes in the appearance of the upper face caused by the action-unit imposter mechanism are sufficient to produce this effect. The current research therefore provides the first evidence to suggest that humans across highly diverse cultural contexts perceive dominance from a downwards head tilt alone, and that they do so at least in part as a result of the action-unit imposter mechanism; this visual illusion might therefore be a universal feature of human cognition.

It is noteworthy that the rate at which participants identified the downwards tilted-head image as dominant was greater when they viewed the whole head, compared to when they were shown only the upper face. This small but significant difference could suggest that additional features of the head, including the mouth, nose, and chin, contribute to perceptions of dominance formed from a downwards head tilt, in this population. However, because participants always viewed the full head image after the upper-face only version, it is also possible that increased dominance perceptions were due merely to repetition and learning. Future research is needed to determine whether additional features of the head contribute to perceptions of dominance formed from a downwards head tilt among the Mayangna, and, if so, whether these features’ role in shaping the social message conveyed by this display is specific to this population.

The present findings make it unlikely that alternative mechanisms previously put forward are primarily responsible for perceptions of dominance formed from a downwards head tilt among the Mayangna. In particular, by showing that these effects emerge when only the upper face is visible, the present findings make it unlikely that the impact of downward-head tilt on dominance perceptions is solely due to: (1) changes in the apparent global dimensions of the head and face (i.e.,^19^, (2) changes in the apparent size of the chin^23^, (3) changes to the appearance of the mouth (e.g.,^25,33,34^); (4) contracting the head towards the body^24^, or (5) covering the neck with the chin^19^.

Nonetheless, the present research cannot completely rule out the possibility that some other visual mechanism accounts for the observed effects. For example, it is possible that Mayangna participants mentally reconstruct the entire head when viewing only the eyes and eyebrows, and their reconstructions include visual changes to other parts of the face— perhaps triggered by seeing V-shaped eyebrows—and these imagined facial components guide their perceptions. Another possibility is that participants use observed changes to the sclera or eyeballs to guide their perceptions. Prior research has ruled out these alternative possibilities in North American samples; even when shown the entire head, precluding imagined reconstructions, and even when eyebrow appearance is manipulated independently of visible sclera (e.g., changes to sclera are methodologically controlled for), V-shaped eyebrows causally predict increased perceptions of dominance and anger from a downwards head tilt^9,11^. Future studies using additional methods and stimuli are needed to test previously proposed alternatives in non-WEIRD populations, to more fully support the universality of the AU imposter effect as the critical mechanism that guides perceptions of dominance from a downwards head tilt.

Future research is also needed to uncover why the action unit imposter mechanism is likely to be a universal feature of human communication. One possibility is that humans evolved to automatically associate perceived geometric angularity in their environments with threat and danger, given that few angular or sharp objects in humans’ ancestral environments did not present a threat to their safety. At some point in human evolutionary history, this adaptive cognitive association might have been co-opted for social communication, such that specific facial expressions, head movements, and bodily behaviors that increase angularity – including the appearance changes to the eyebrows that occur when the head is tilted down –also guide human threat communication (Witkower & Tracy, under review). If the association between a downwards head tilt and threat co-opts a broader and evolved association between angularity and threat, both associations could be universal.
